# Multi-omics analysis reveals the phenylalanine pathway associated with stem development under organic and compound fertilizers in *Setaria italica*

**DOI:** 10.3389/fpls.2026.1899229

**Published:** 2026-08-07

**Authors:** Jiujun Du, Yongxia Bai, Zhuohan Gao, Yu Zhao, Jihan Cui, Min Zhang, Fengyan Cao, Meihong Huang, Shunguo Li, Xueyan Xia

**Affiliations:** 1 Institute of Millet Crops, Hebei Academy of Agriculture and Forestry Sciences/Key Laboratory of Genetic Improvement and Utilization for Featured Coarse Cereals (Co-Construction by Ministry and Province), Ministry of Agriculture and Rural Affairs/The Key Research Laboratory of Minor Cereal Crops of Hebei Province, Shijiazhuang, China; 2 State Key Laboratory of Tree Genetics and Breeding, Key Laboratory of Tree Breeding and Cultivation of National Forestry and Grassland Administration, Research Institute of Forestry, Chinese Academy of Forestry, Beijing, China

**Keywords:** cell wall, cow dung, foxtail millet, multi-omics, phenylalanine

## Abstract

**Introduction:**

To explore the mechanism underlying the effects of combined application of organic fertilizer and compound fertilizer on stem development of foxtail millet, this study used ‘*Jiza Jinmiao 3*’ as the test material and set up three treatments: sole application of organic manure (Control1), sole application of compound fertilizer (Control2), and combined application of cattle manure-based organic fertilizer and compound fertilizer (Treatment).

**Methods:**

The effects of different fertilizer treatments were analyzed by integrating phenotypic determination, microscopic observation of stem structure, and multi-omics analyses including transcriptomics, metabolomics, and proteomics.

**Results:**

The results showed that compared with the sole application treatments, the combined application treatment significantly promoted phenotypic indicators of foxtail millet such as plant height, stem diameter, and stem fresh weight, and also significantly increased the cross-sectional area of stem vessels, the cell wall thickness of basic tissues, and the stem puncture strength. Multi-omics analyses revealed that differentially expressed genes (DEGs), differentially accumulated metabolites (DAMs), and differentially expressed proteins (DEPs) under the combined application treatment were significantly enriched in pathways related to cell wall development, such as phenylalanine metabolism and phenylpropanoid biosynthesis. Specifically, mapped to the phenylalanine pathway, the expression levels of key genes, metabolites, and proteins including *Phenylalanine Ammonia*-*Lyase* (*PAL*), *Phenylalanine*/*Tyrosine Ammonia*-*Lyase* (*PTAL*), and *Peroxidase* gene (*PRDX6*) were significantly upregulated, and the content of phenylalanine was increased.

**Discussion:**

This study suggests that the combined application of organic fertilizer and compound fertilizer is associated with enhanced stem structural properties (including increased vascular area, cell wall thickness, and stem puncture strength) potentially linked to improved lodging resistance, with the phenylalanine pathway emerging as a candidate pathway, providing a theoretical basis and technical support for optimizing fertilization strategies and improving yield stability in the high-yield cultivation of foxtail millet.

## 1 Introduction

Foxtail millet (*Setaria italica*), an important minor cereal crop in northern China, possesses characteristics of barren tolerance, high nutritional value (rich in high-quality protein and dietary fiber), and adaptability to marginal croplands (Gao et al., 2020; Department of Rural Surveys, N.S.O.C., 2023; Qu et al., 2023). It plays a crucial role in safeguarding the diversification of the grain structure and promoting the sustainable development of regional agriculture (Li and Brutnell, 2011; Ni et al., 2017; Pan et al., 2018; Ma et al., 2023; Rajyaguru et al., 2024).

Fertilization is a key agronomic measure for regulating crop stem growth. Farmyard manures such as composted cow dung can improve soil physical and chemical properties and release organic matter slowly, providing long-term environmental support for the entire growth period of foxtail millet (Klinger et al., 2013; Zavattaro et al., 2016; Pan and Huang, 2024; Zhou et al., 2025); compound fertilizers (inorganic fertilizers) can quickly supplement essential mineral elements such as nitrogen, phosphorus, and potassium, precisely meeting the nutrient requirements of foxtail millet (Adesemoye et al., 2009). However, single application of farmyard manure has the problem of limited short-term growth-promoting effects, while single application of compound fertilizers tends to cause nutrient imbalance. Moreover, unreasonable application of chemical fertilizers can lead to soil salinization. In contrast, the ‘synergistic effect’ potential of their combined application has gradually become prominent (Yang et al., 2005; Rengasamy, 2006; Wan et al., 2021). Nevertheless, research on the combined application of organic and compound fertilizers in crops has mostly focused on crop yield, water use, and soil improvement (Chen et al., 2023; Shang et al., 2025; Yang et al., 2025). Studies on plant stem development and cell walls are relatively weak, lacking specialized analysis (especially microstructures such as vascular bundles and cell walls). Additionally, no clear correlation among “fertilization treatment-changes in stem microstructure-phenotypic growth” has been established, making it impossible to explain the physiological process by which combined fertilization promotes stem development.

With the rapid advancement of high-throughput sequencing technology, multi-omics integrated analysis has emerged as a powerful tool for deciphering the associations between plant phenotypes and molecular regulatory networks. However, systematic exploration of differentially expressed genes (DEGs), differentially accumulated metabolites (DAMs), and differentially expressed proteins (DEPs) related to foxtail millet stem development under the combined application of farmyard manure and compound fertilizer remains lacking, particularly via the integrated ‘transcriptomics + metabolomics + proteomics’ approach. In this study, the foxtail millet cultivar ‘*Jiza Jinmiao 3*’ was used as the experimental material, with three fertilization treatments established: sole application of composted cow dung (Control1), sole application of compound fertilizer (Control2), and combined application of composted cow dung and compound fertilizer (Treatment). The objectives of this research were twofold: first, to clarify the regulatory effects of combined fertilization on the phenotypic traits and microstructure of foxtail millet stems, and verify whether the combined application outperforms single fertilization regimes; second, to screen DEGs, DAMs, and DEPs associated with stem development under the combined fertilization treatment, thereby identifying core regulatory pathways and revealing the molecular mechanisms underlying the promotion of foxtail millet stem development by the combined application of farmyard manure and compound fertilizer. Theoretically, this study will enrich the research field of “the regulatory mechanisms of organic and inorganic fertilization on foxtail millet stem growth” at the genetic, metabolomic, and proteomic levels, clarify the potential association between fertilization regimes and stem structural properties relevant to lodging resistance, and provide a theoretical basis for improving the yield and economic benefits of foxtail millet.

## 2 Materials and methods

### 2.1 Plant materials and fertilization treatments

The experiment utilized foxtail millet inbred variety ‘*Jiza Jinmiao 3*’, sown in 2024 and 2025 at the Mazhuang experimental station (37°93’ N, 114°78’ E) in Gaocheng District, Shijiazhuang City. The field experiment was conducted using a randomized complete block design with three replications. Three treatments were implemented: Control1 (582.68 g/m² of decomposed cattle manure), Control2 (59.97 g/m² of compound fertilizer (N-P_2_O_5_-K_2_O=27-8-6, Batian, Shenzhen, China)), and Treatment (425.79 g/m² decomposed cattle manure + 44.98 g/m² compound fertilizer). Total amounts of total N, total P_2_O_5_ and total K_2_O were kept identical across all treatments based on measured total N, total P and total K concentrations of organic manure. Urea, superphosphate and potassium sulfate were supplemented to equalize N, P and K inputs for each treatment, respectively (**Table 1**). All other cultivation practices followed standard operational procedures during the growing season.

**Table 1:** Nutrient composition of organic manure.

Item	Total N (%)	Total P (g/kg)	Total K (g/kg)
Organic manure	0.961	5.93	17.5

### 2.2 Phenotypic data measurement

Growth parameters were measured at the jointing stage, including plant height, stem basal diameter, fresh/dry stem weight, and fresh/dry leaf weight. Concurrently, stem sections were prepared using a microtome LEICA VT1000 at a thickness of 40 μm for quantification of vascular cross-sectional area and cell wall thickness (Leica Microsystems, Germany). All phenotypic parameters were measured using six biological replicates per treatment group (n = 6). All measured data were obtained from representative samples of each experimental plot, and sampling was performed with the exclusion of border rows to eliminate edge effects.

### 2.3 Omics analysis

At the jointing stage of foxtail millet, stem segment samples from the seventh internode of uniformly growing plants were collected, immediately frozen in liquid nitrogen, and stored at -80 °C before being sent to Shanghai Majorbio Bio-Pharm Technology Co., Ltd. (Shanghai, China) for multi-omics analysis including transcriptomics, metabolomics, and proteomics. The reference genome used in the analyses was *Setaria italica* v2.2. The analysis of omics data was performed on Majorbio Cloud (Ren et al., 2022). For transcriptomics, differentially expressed genes (DEGs) were identified using |log2 fold change| > 1 and Benjamini-Hochberg adjusted *p*-value (FDR) < 0.05. For metabolomics, differentially accumulated metabolites (DAMs) were selected based on Partial Least Squares Discriminant Analysis (PLS-DA) with 95% confidence level and 200 permutation tests. For proteomics, differentially expressed proteins (DEPs) were identified using *p*-value < 0.05 and fold change > 1.2 (upregulated) or < 0.83 (downregulated). For transcriptomic and proteomic analyses, three biological replicates per treatment group (n = 3) were used. For widely targeted metabolomics, six technical replicates per treatment group (n = 6) were analyzed. The phenylalanine and indole-3-acetic acid (IAA) content in the stems of foxtail millet at the jointing stage was determined by Wuhan Metware Biotechnology Co., Ltd, three biological replicates per treatment group (n = 3) were used.

### 2.4 Primer design and qPCR assays

To validate the gene expression levels obtained from the transcriptome results, six genes were randomly selected for qPCR primer design using Primer3Plus (https://www.primer3plus.com/). The qPCR experiment was conducted on the CFX Opus Deepwell (Bio-rad, USA) (**Supplementary Table 1**). The 2^-ΔΔCT^ method and *Actin* gene (Seita.7G294000) (Dai et al., 2024) were employed to analyze the relative expression levels. qPCR was performed with three biological replicates per treatment group (n = 3). For correlation analysis between RNA-seq and qPCR data, expression values of each gene were first normalized to z-scores within each gene to enable cross-platform comparison. Pooled z-score normalized data across all ten genes (n = 90 data points) were used to calculate the overall Pearson correlation coefficient (*r*) and two-tailed *p*-value using Fisher’s z-transformation. A scatter plot of pooled z-scores was generated with a linear regression line (dashed), and the *r* and *p* values were annotated on the plot. Per-gene Pearson correlation coefficients were also calculated independently (n = 9 per gene) and visualized as a horizontal bar chart. All correlation analyses and visualizations were performed in Python using NumPy, SciPy, and Matplotlib.

### 2.5 Multi-omics correlation network analysis

Multi-omics correlation network analysis: Pairwise Pearson correlation coefficients were calculated for all 51 molecules (8 DEGs, 8 DAMs, and 35 DEPs) mapped to the phenylalanine/phenylpropanoid pathway. Each molecule was first z-score standardized (mean = 0, SD = 1) across the nine sample points (3 treatments × 3 biological replicates). For metabolomics data, the six technical replicate measurements per treatment were averaged in pairs (replicates 1-2, 3-4, 5-6) to yield three biological replicates consistent with the transcriptomic and proteomic experimental design. Correlation significance was assessed using two-tailed p-values. Integrative pathway enrichment scores for the phenylpropanoid biosynthesis and phenylalanine metabolism pathways were computed by combining the KEGG enrichment p-values from the three omics layers using Fisher’s method (chi-square = -2 x sum ln(*p*), df = 6). Correlation heatmaps were generated using Python (NumPy, SciPy, Matplotlib).

### 2.6 Data analysis

Data were analyzed using one-way ANOVA in SPSS 29 (IBM, USA), with statistical significance set at *p* < 0.05. *Post-hoc* Tukey’s test was applied for multiple comparisons if significant differences were detected. Values are expressed as Mean ± SE. Graphs were generated with GraphPad Prism 10 (GraphPad Software, USA).

## 3 Results

### 3.1 Synergistic effects of farmyard manure and compound fertilizer

**Figure 1:**
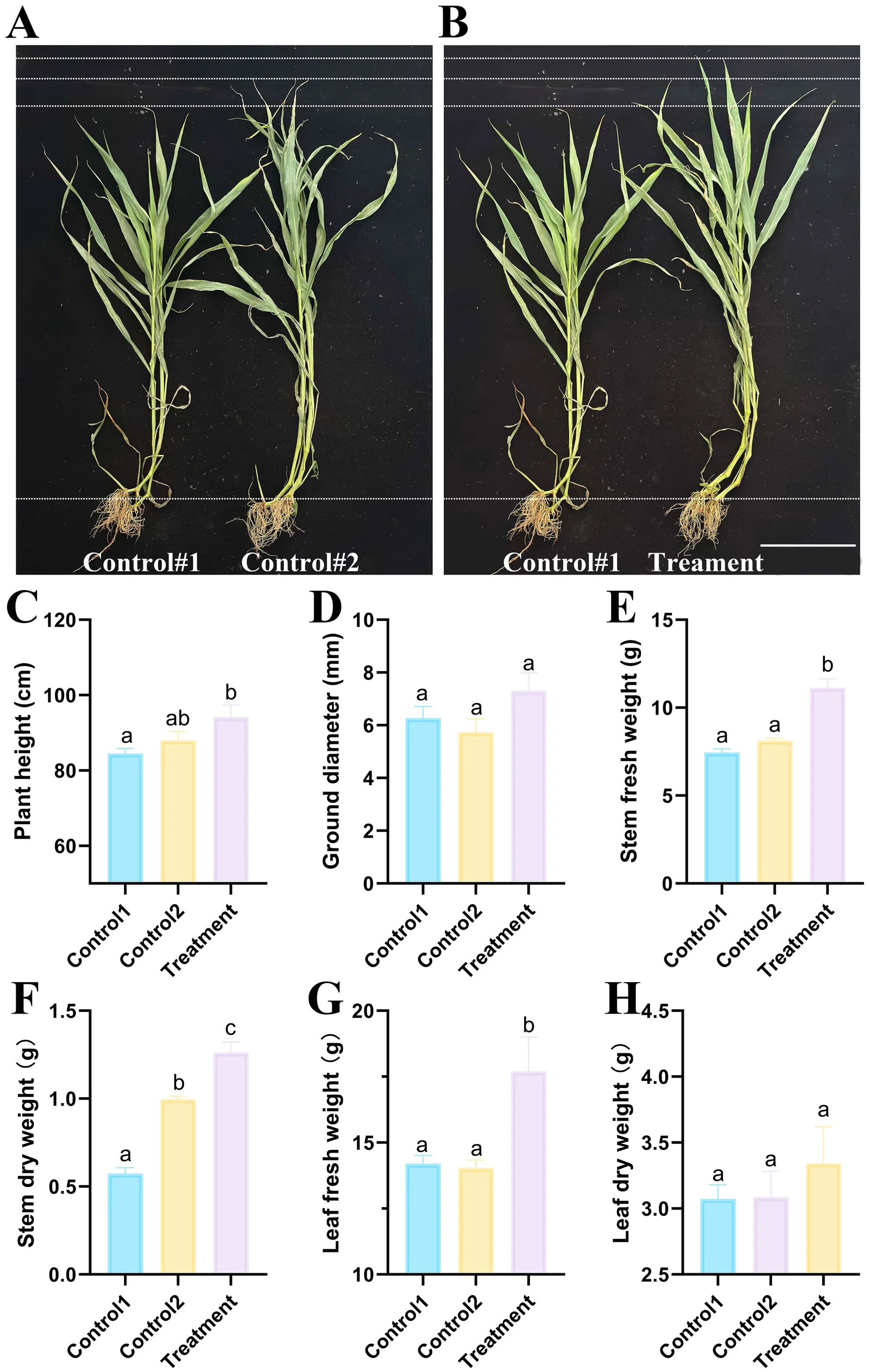
Phenotypic parameter measurements. **(A, B)** General phenotype of *Setaria italica*. Bar = 20 cm. **(C)** Plant height. **(D)** Ground diameter. **(E)** Stem fresh weight. **(F)** Stem dry weight. **(G)** Leaf fresh weight. **(H)** Leaf dry weight. Data are presented as means ± SE (n = 6), and the weight-related traits (stem fresh weight, stem dry weight, leaf fresh weight, leaf dry weight) were measured with six biological replicates per treatment; replicate pairs were averaged (replicates 1–2, 3–4, 5–6) to yield n = 3 independent values for statistical analysis. Different letters above bars indicate significant differences among treatments (one-way ANOVA with Dunnett’s test, *p* < 0.05).

Compared with the sole application of cow dung, the application of compound fertilizer promoted plant development. Meanwhile, the combined application of the two fertilizers enhanced plant phenotypes compared with the sole application of either fertilizer (**Figures 1A, B**). Determination of aboveground growth indicators also showed that the combined application of compound fertilizer and cow dung promoted the stem growth of foxtail millet. Specifically, the plant height, stem weight, and leaf fresh weight under the combined application were significantly higher than those under the sole application of cow dung, while the ground diameter and leaf dry weight were also higher than those under the sole application of cow dung (**Figures 1C–H**). Furthermore, consistent trends were observed in our measurements and analyses conducted in 2025 (**Supplementary Figure 1**).

**Figure 2:**
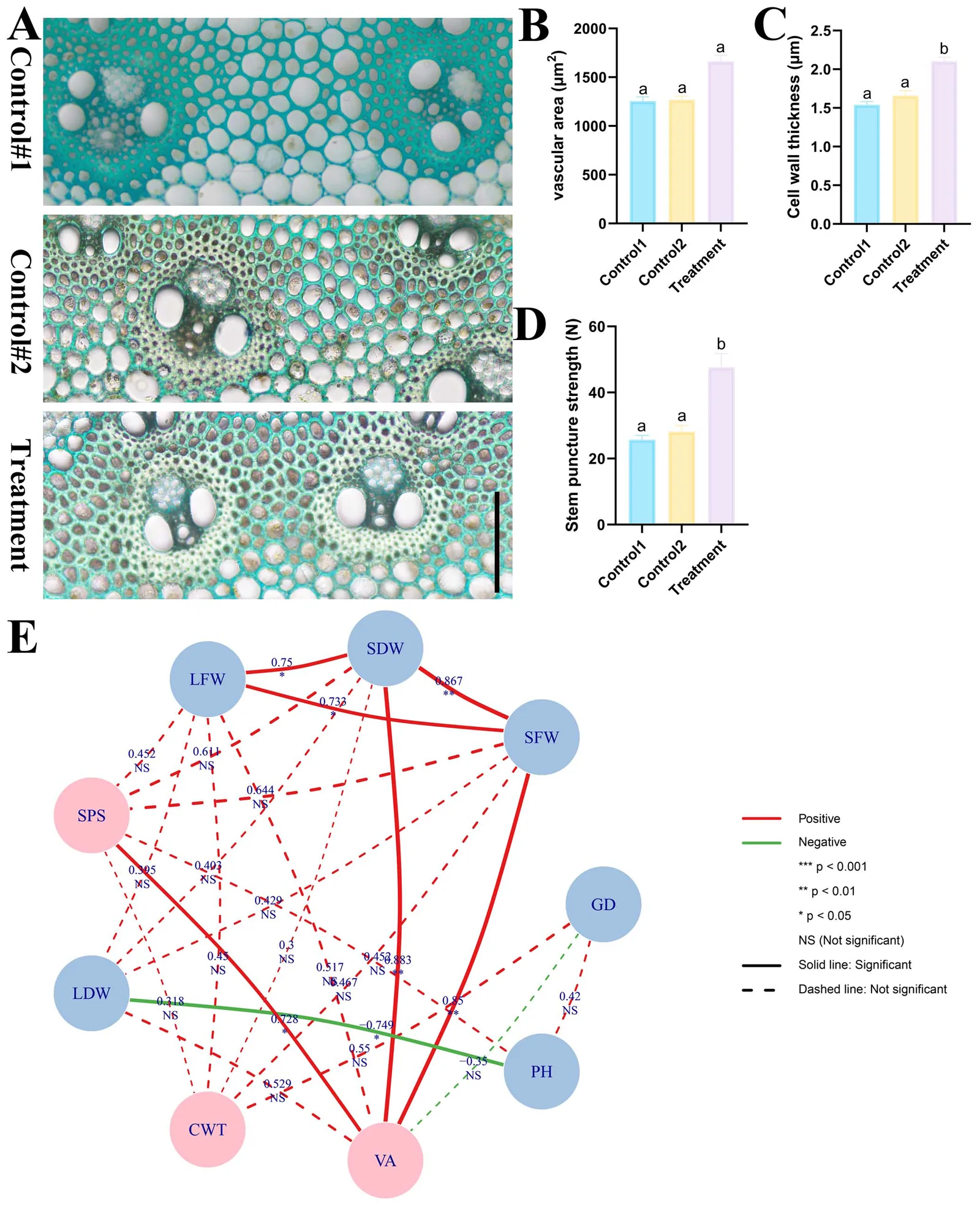
Observation and analysis of stem microstructure. **(A)** General phenotype of vascular bundle development in foxtail millet stems. Statistical analysis was conducted on vascular area **(B)**, cell wall thickness **(C)**, and stem puncture strength **(D)**. n = 3, *p*-values obtained by one-way ANOVA for genotype and the bars indicate means ± SE, with different letters on the error bars denote significant differences among samples. Scale bar = 100 μm. **(E)** The figure shows the correlation relationships among 9 phenotypic traits, including plant height (PH), ground diameter (GD), vessel cross-sectional area (VA), cell wall thickness (CWT), stem penetration strength (SPS), stem fresh weight (SFW), stem dry weight (SDW), leaf fresh weight (LFW), and leaf dry weight (LDW). Lines of different colors indicate positive or negative correlations, with correlation coefficients labeled on the lines; *p*-values are marked with asterisks (*p < 0.05, **p < 0.01, ***p < 0.001), and solid and dashed lines represent different conditions or significance levels.

Given the effects of combined fertilization on stem-related traits, the fourth internode of foxtail millet was selected for stem microstructure analysis. The results showed that the combined application treatment induced changes in vessel elements and ground tissue (**Figure 2A**). Specifically, the cross-sectional area of vessels was significantly larger than that under the sole application of either fertilizer. Meanwhile, the cell wall thickness of ground tissue also changed consistently with the vessel alterations, being significantly greater under combined fertilization compared to the sole application of each fertilizer (**Figures 2B,C**). Since cell wall development affects stem physical strength (Hongo et al., 2012), we determined the stem puncture strength across different treatments. Consistent with expectations, the stem puncture strength under combined fertilization was significantly higher than those of the two control groups (**Figure 2D**). Correlation analysis between microstructure and phenotypic traits revealed that vessel area was significantly positively correlated with the fresh weight, dry weight of stem segments, and stem puncture strength, with correlation coefficients of 0.883 (**), 0.85 (*), and 0.728 (*), respectively. These results suggested that vessel development promotes stem growth and enhances stem physical strength (**Figure 2E**).

### 3.2 Analysis of differentially expressed genes

To clarify the molecular mechanism of different fertilizer response, transcriptomic sequencing was performed on the stems of plants from the three treatment groups. The number of clean reads ranged from 39,845,136 to 46,241,516, with Q30 values exceeding 95% (sequencing error rate < 0.13%) and alignment rates to the reference genome higher than 91.81% (**Supplementary Tables 2**, **3**), confirming high data quality. Principal component analysis (PCA) and sample correlation heatmaps showed distinct inter-group separation and high intra-group correlation (**Supplementary Figures 2A, B**). qPCR was performed to validate the expression profiles of randomly selected genes, which ensured the accuracy of the transcriptome sequencing data (**Supplementary Figure 3**).

**Figure 3:**
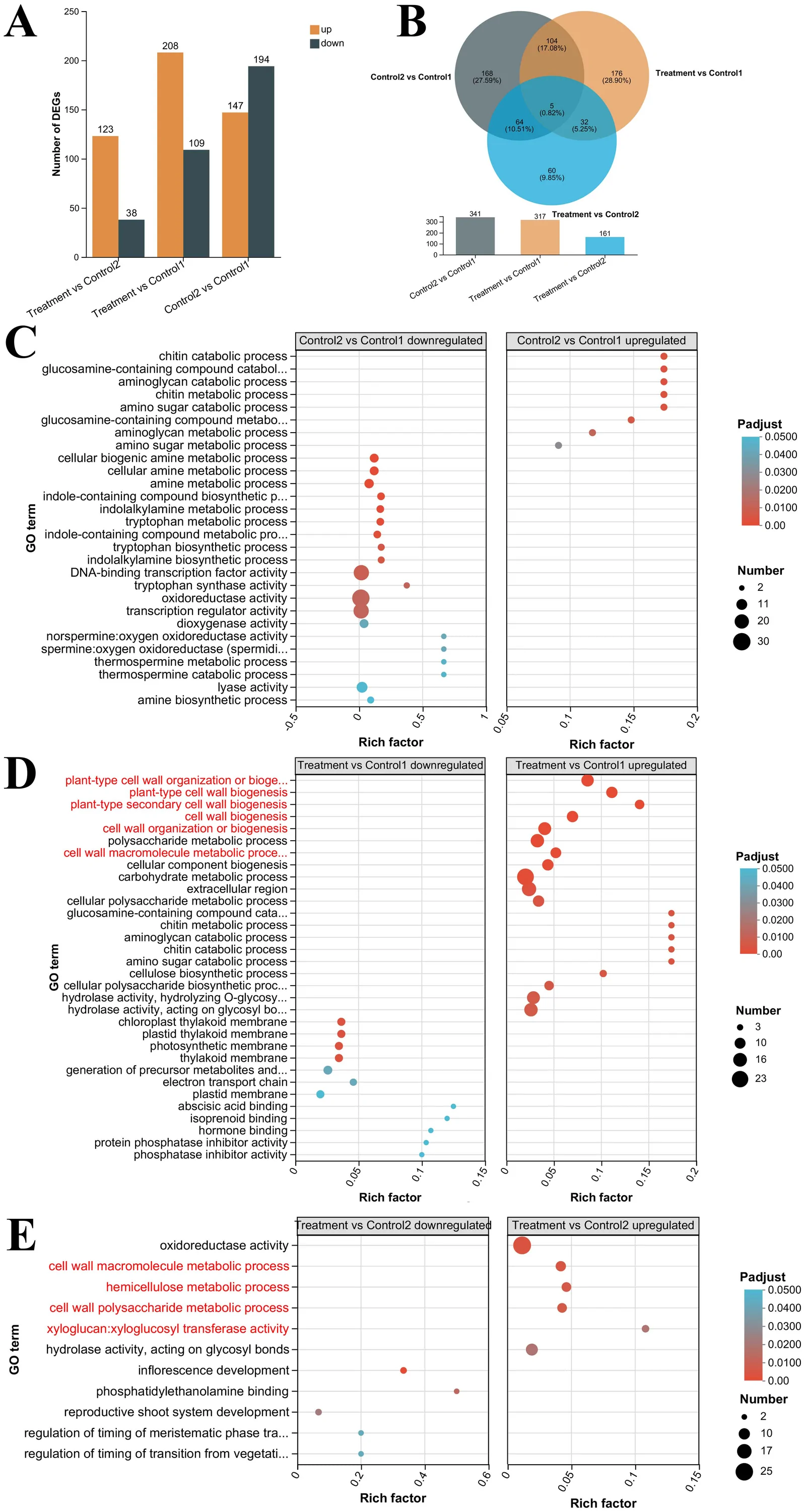
Analysis of DEGs. **(A)** Statistical bar chart of the number of DEGs in each comparison group; bars of different colors represent upregulated and downregulated DEGs, respectively. **(B)** Venn diagram of DEGs in each comparison group, with the total number of DEGs in each comparison group counted. **(C–E)** GO enrichment results of the Control2 vs Control1, Treatment vs Control1, and Treatment vs Control2 comparison groups, respectively. In the figures, the color of the bubbles indicates the statistical significance of the enriched pathways, and the size of the bubbles represents the number of DEGs contained in the corresponding pathways. n = 3 biological replicates per treatment group.

In the Control2 vs Control1 comparison group, 147 differentially expressed genes (DEGs) were upregulated and 194 were downregulated, totaling 341 DEGs, which was the group with the largest number of DEGs. Followed by the Treatment vs Control1 group, where 208 DEGs were upregulated and 109 were downregulated, totaling 317. The Treatment vs Control2 group had the fewest DEGs (161 in total), including 123 upregulated and 38 downregulated genes (**Figures 3A, B**). Gene Ontology (GO) enrichment analysis of DEGs showed that the upregulated DEGs in the Treatment vs Control1 group were significantly enriched in 8 terms (adjusted *p*-value < 0.05), all related to carbohydrate metabolism, specifically chitin and amino sugar catabolic processes (e.g., chitin catabolic/metabolic process, glucosamine-containing compound/aminoglycan/amino sugar catabolic process). In contrast, the downregulated DEGs were significantly enriched in 21 terms (adjusted p-value < 0.05), covering core biological processes and molecular functions such as amine metabolism, tryptophan metabolism, indole-containing compound biosynthesis, transcriptional regulation, and enzyme activity (e.g., amine metabolic process, tryptophan biosynthetic/metabolic process, indole-containing compound biosynthetic/metabolic process, transcription regulator activity) (**Figure 3C**; **Supplementary Tables 4**, **5**).

In the Treatment vs Control1 comparison group, the upregulated DEGs were significantly enriched in 17 terms, covering plant cell wall biogenesis, polysaccharide metabolism, and aminoglycan/amino sugar catabolic processes (e.g., cell wall biogenesis, polysaccharide metabolic process, aminoglycan catabolic process, amino sugar catabolic process). In contrast, the downregulated DEGs were significantly enriched in 11 terms, involving key biological processes such as photosynthetic structure, energy metabolism, hormone binding, and enzyme activity regulation (e.g., plastid thylakoid membrane, photosynthetic membrane, generation of precursor metabolites and energy, hormone binding, protein phosphatase inhibitor activity) (**Figure 3D**; **Supplementary Tables 6**, **7**).

In the Treatment vs Control2 comparison group, the upregulated DEGs were significantly enriched in 6 terms, focusing on two core areas: enzyme activity regulation and plant cell wall metabolism (e.g., oxidoreductase activity, cell wall macromolecule metabolic process, cell wall polysaccharide/hemicellulose metabolic process). In contrast, the downregulated DEGs were significantly enriched in 5 terms, related to plant reproductive development regulation and molecular binding functions (e.g., inflorescence development, reproductive shoot system development, phosphatidylethanolamine binding) (**Figure 3E**; **Supplementary Tables 8**, **9**). Combined with the phenotypic analysis results, the upregulated DEGs in the Treatment vs Control1 group were mostly enriched in plant cell wall-related pathways, with 6 terms significantly enriched. Similar results were observed in the Treatment vs Control2 group, where 4 terms were associated with plant cell wall development. These findings suggest that the application of compound fertilizer may promote plant cell wall development, and the combined application of farmyard manure and compound fertilizer exhibits a more pronounced promoting effect on cell wall development compared to the sole application of compound fertilizer.

**Figure 4:**
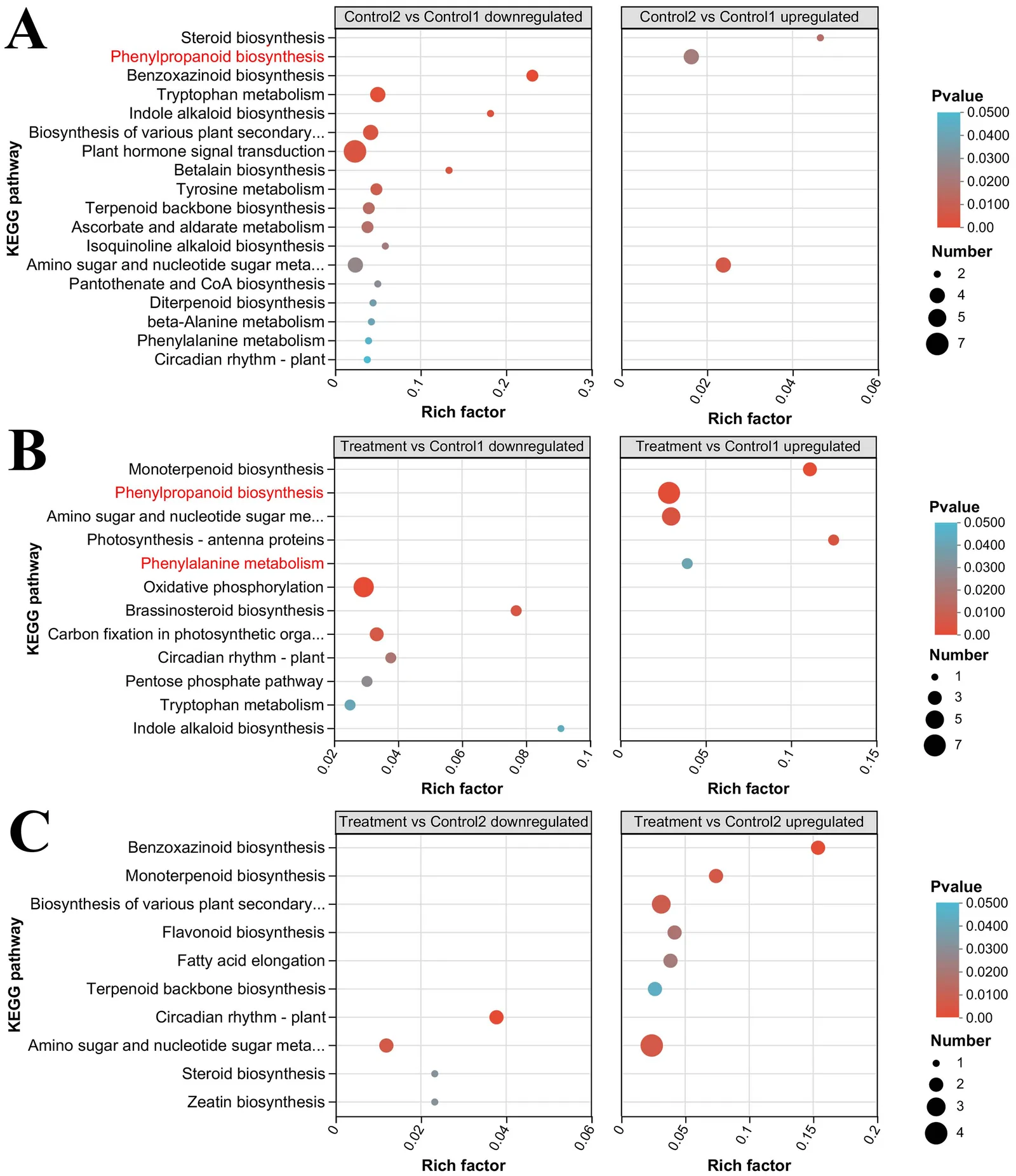
KEGG enrichment analysis of DEGs. **(A–C)** represent the KEGG enrichment results of the comparison groups Control2 vs Control1, Treatment vs Control1, and Treatment vs Control2, respectively. In the figure, the color of the bubbles indicates the statistical significance of the enriched pathways, and the size of the bubbles represents the number of DEGs contained in the corresponding pathways. n = 3 biological replicates per treatment group.

Kyoto Encyclopedia of Genes and Genomes (KEGG) enrichment analysis was further performed on the DEGs, and the results were generally consistent with those of GO enrichment analysis. In the Control2 vs Control1 and Treatment vs Control1 comparison groups, the upregulated DEGs were also significantly enriched in the Phenylpropanoid biosynthesis and Phenylalanine metabolism pathways (*p* < 0.05). In contrast, the downregulated DEGs in the two groups were enriched in 5 pathways related to secondary metabolic pathways and hormone signal regulation, and pathways involving plant energy conversion, carbon metabolism, hormone synthesis, and rhythmic regulation, respectively (**Figures 4A, B**; **Supplementary Tables 10**–**13**).

In the Treatment vs Control2 comparison group, the upregulated DEGs were significantly enriched in 7 metabolic pathways, including secondary metabolite synthesis, carbohydrate metabolism, lipid synthesis, and terpenoid backbone biosynthesis. In contrast, the downregulated DEGs were enriched in 4 pathways: Circadian rhythmplant, Amino sugar and nucleotide sugar metabolism, Steroid biosynthesis, and Zeatin biosynthesis (**Figure 4C**; **Supplementary Tables 14**, **15**). After the combined application of compound fertilizer and cow dung, the upregulated DEGs were significantly enriched in the phenylalanine synthesis and metabolism pathways, suggesting that the combined application of the two fertilizers affects the morphogenesis of plant cell walls.

To validate the transcriptomic data, qPCR was performed on ten genes, including six key nodes in the phenylalanine/phenylpropanoid pathway and four additional differentially expressed genes. Expression values of each gene were z-score normalized to enable cross-platform comparison. The pooled z-score normalized Pearson correlation between RNA-seq and qPCR across all 10 genes (n = 90 data points) was *r* = 0.249 (*p* = 0.018, two-tailed test using Fisher’s z-transformation), indicating overall concordance between the two platforms. Per-gene Pearson correlation coefficients ranged from r = -0.218 (*trpC*) to *r* = 0.571 (*PTAL2*) (n = 9 per gene); although no individual gene reached statistical significance at *p* < 0.05, the overall positive trend supports the reliability of the transcriptomic quantification. A correlation scatter plot and per-gene bar chart are provided as **Supplementary Figure 4**.

**Figure 5:**
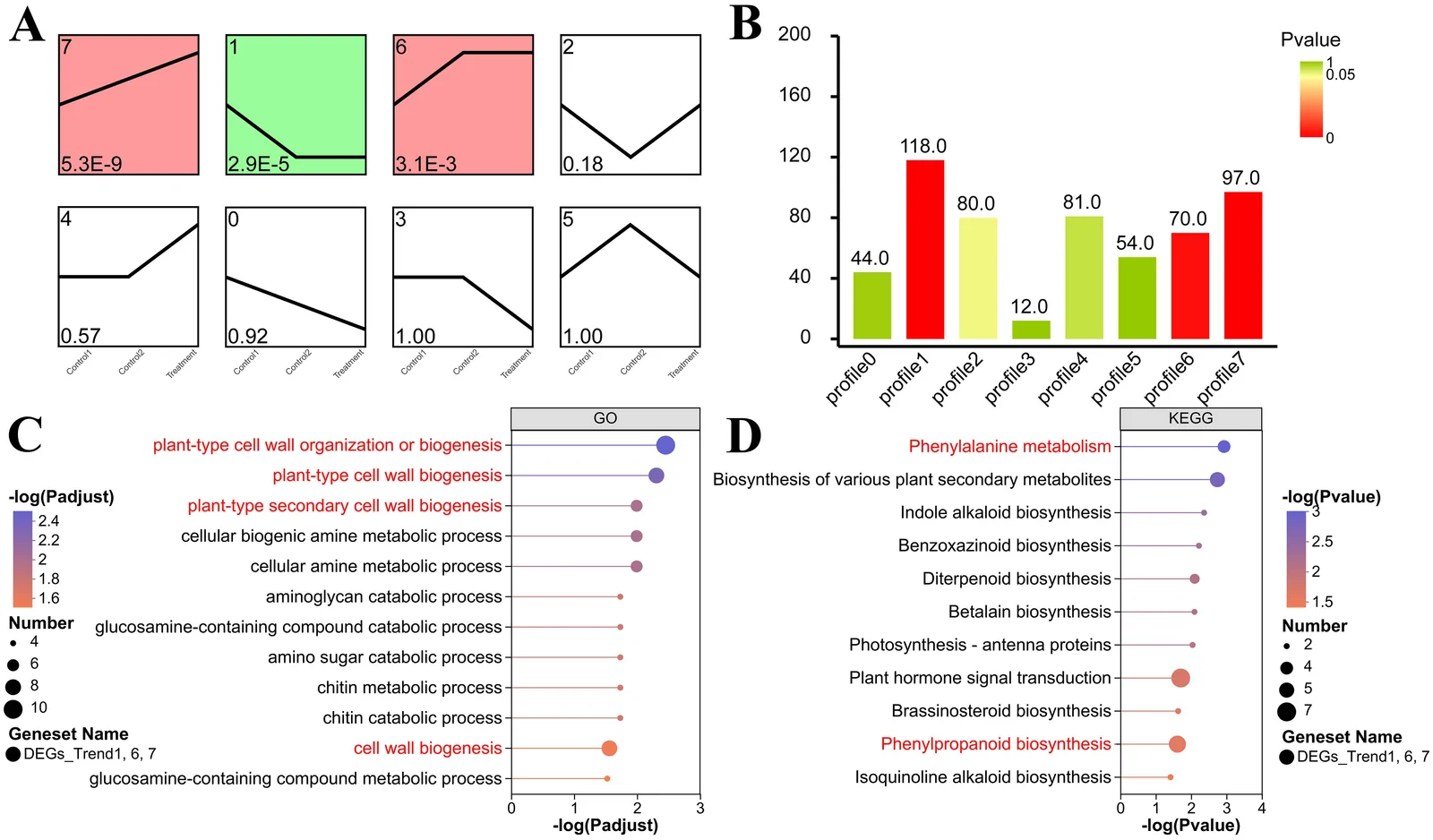
Trend analysis of DEGs. **(A)** The DEGs were significantly enriched into 3 trends, and the *p*-values of each trend are marked in the figure. **(B)** The DEGs contained in each trend, and the color of the column chart represents the p-value of each trend. **(C, D)** GO and KEGG enrichment analysis of DEGs in trend 1, 6 and 7. The color of the bubbles in the figure represents the statistical significance of the enriched pathway, and the size of the bubbles represents the number of DEGs contained in the corresponding pathway. n = 3 biological replicates per treatment group.

Trend enrichment analysis was further performed on the DEGs. A total of 609 DEGs were enriched into 8 trends, among which Trend 1 (*p* = 5.3E-9), Trend 6 (*p* = 2.9E-5), and Trend 7 (*p* = 3.1E-3) were significantly enriched. These three trends contained 118, 97, and 70 DEGs respectively, with a relatively large number of genes. Trends 6 and 7 were positively correlated with growth phenotypes, while Trend 1 was negatively correlated with growth phenotypes, suggesting that the DEGs enriched in Trends 1, 6, and 7 may be involved in the phenotypic regulation of foxtail millet (**Figures 5A, B**; **Supplementary Table 16**). Subsequently, separate GO and KEGG enrichment analyses were conducted on the 285 DEGs enriched in Trends 1, 6, and 7. GO enrichment analysis showed that the DEGs were significantly enriched in 12 terms (adjusted p-value < 0.05), among which 4 terms were related to cell wall development, and 3 of these 4 terms had the smallest adjusted *p*-value. KEGG enrichment analysis revealed that the DEGs were significantly enriched in 11 pathways (*p* < 0.05), with the phenylalanine metabolism pathway having the smallest *p*-value, and the Phenylpropanoid biosynthesis pathway also being significantly enriched. The trend and enrichment results indicated that most DEGs were associated with plant cell wall development, which was consistent with the transcriptomic analysis results in **Figure 3** (**Supplementary Tables 17**, **18**).

### 3.3 Analysis of differentially accumulated metabolites

**Figure 6:**
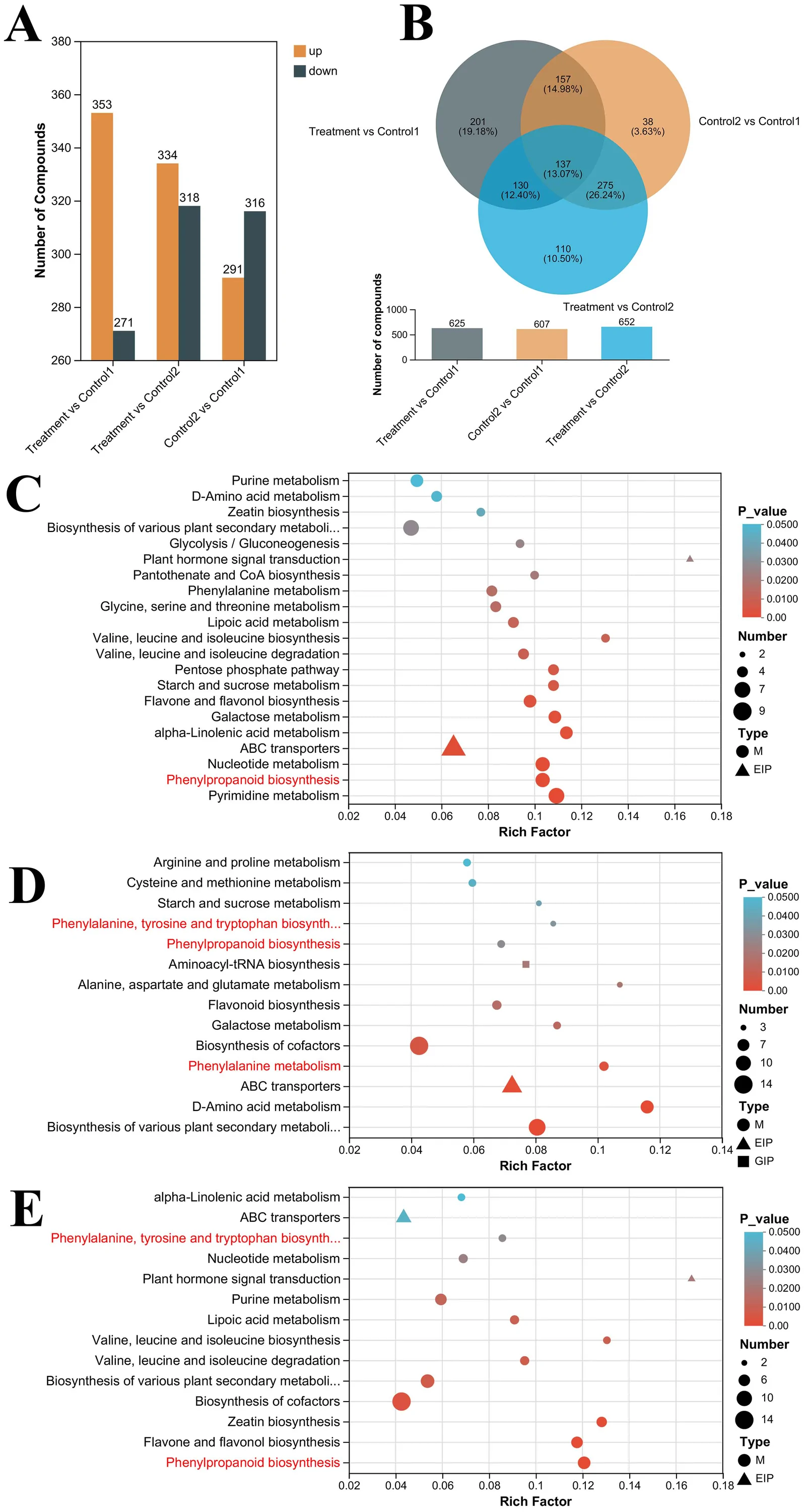
Analysis of DAMs. **(A)** Bar chart showing the number of DAMs in each comparison group, where colors of different bars represent upregulated and downregulated DAMs, respectively. **(B)** Venn diagram of DAMs among comparison groups, with the total number of DAMs in each group counted. **(C–E)** KEGG enrichment analysis of DAMs. In the figures, the color of bubbles indicates the statistical significance of the enriched pathways, the size of bubbles represents the number of DAMs contained in the corresponding pathways, and the shapes (circle, triangle, and square) represent the categories of Metabolism, Environmental Information Processing, and Genetic Information Processing, respectively. n = 6 biological replicates per treatment group.

To investigate the effects of different fertilization treatments on the metabolites of foxtail millet, a widely targeted metabolomics study was conducted using mass spectrometry. PCA and sample correlation analysis showed distinct inter-group separation and strong intra-group reproducibility (**Supplementary Figures 5A, B**). Orthogonal partial least squares discriminant analysis (OPLS-DA) revealed R^2^Y = 0.999 and Q^2^ > 0.99 in all comparisons, confirming the high accuracy of the model (**Supplementary Figure 5C**). A total of 1421 and 937 metabolites were identified in positive and negative ion modes, respectively, with 1415 and 935 metabolites retained after filtering (**Supplementary Tables 19**, **20**). These metabolites were mainly classified into 357 lipids and lipid-like molecules, 295 phenylpropanoids and polyketides, 267 organic acids and their derivatives, 218 organoheterocyclic compounds, and 208 organooxygen compounds (**Supplementary Figure 5D**). A total of 1048 differential accumulated metabolites (DAMs) were detected across the three comparison groups, with 652, 625, and 607 DAMs enriched in Control2 vs Control1, Treatment vs Control1, and Treatment vs Control2, respectively. The overall trend in the number of DAMs was consistent with that of DEGs (**Figures 6A, B**; **Supplementary Figures 5E–G**). Among the top 60 DAMs (ranked by Variable Importance in Projection (VIP) values) in all comparison groups, phenylalanine accounted for the highest proportion in Treatment vs Control1 (22%) and Treatment vs Control2 (24%), and the second highest in Control2 vs Control1 (18%) (**Supplementary Figure 6**).

In the Control2 vs Control1 comparison group, DAMs were enriched in 21 core pathways, including plant primary metabolism (e.g., carbohydrates, amino acids, nucleotides), secondary metabolism (e.g., phenylpropanoids, flavonoids), substance transport, and signal transduction. Among these, two pathways — Phenylpropanoid biosynthesis and Starch and sucrose metabolism—are associated with plant cell wall development (**Figure 6C**; **Supplementary Table 21**). In the Treatment vs Control1 group, 5 out of the 14 enriched pathways were related to plant cell wall development, including three phenylalanine-related pathways and two carbohydrate metabolic pathways (**Figure 6D**; **Supplementary Table 22**). For the Treatment vs Control2 group, DAMs were enriched in 14 core pathways involving plant secondary metabolism, amino acid metabolism, nucleotide metabolism, hormone signaling, and substance transport, which also included two phenylalanine metabolism-related pathways (**Figure 6E**; **Supplementary Table 23**). Across the three comparison groups, the Treatment vs Control1 group contained the most cell wall development-related pathways (5 in total), which was consistent with the transcriptomic results.

### 3.4 Analysis of differentially expressed proteins

**Figure 7:**
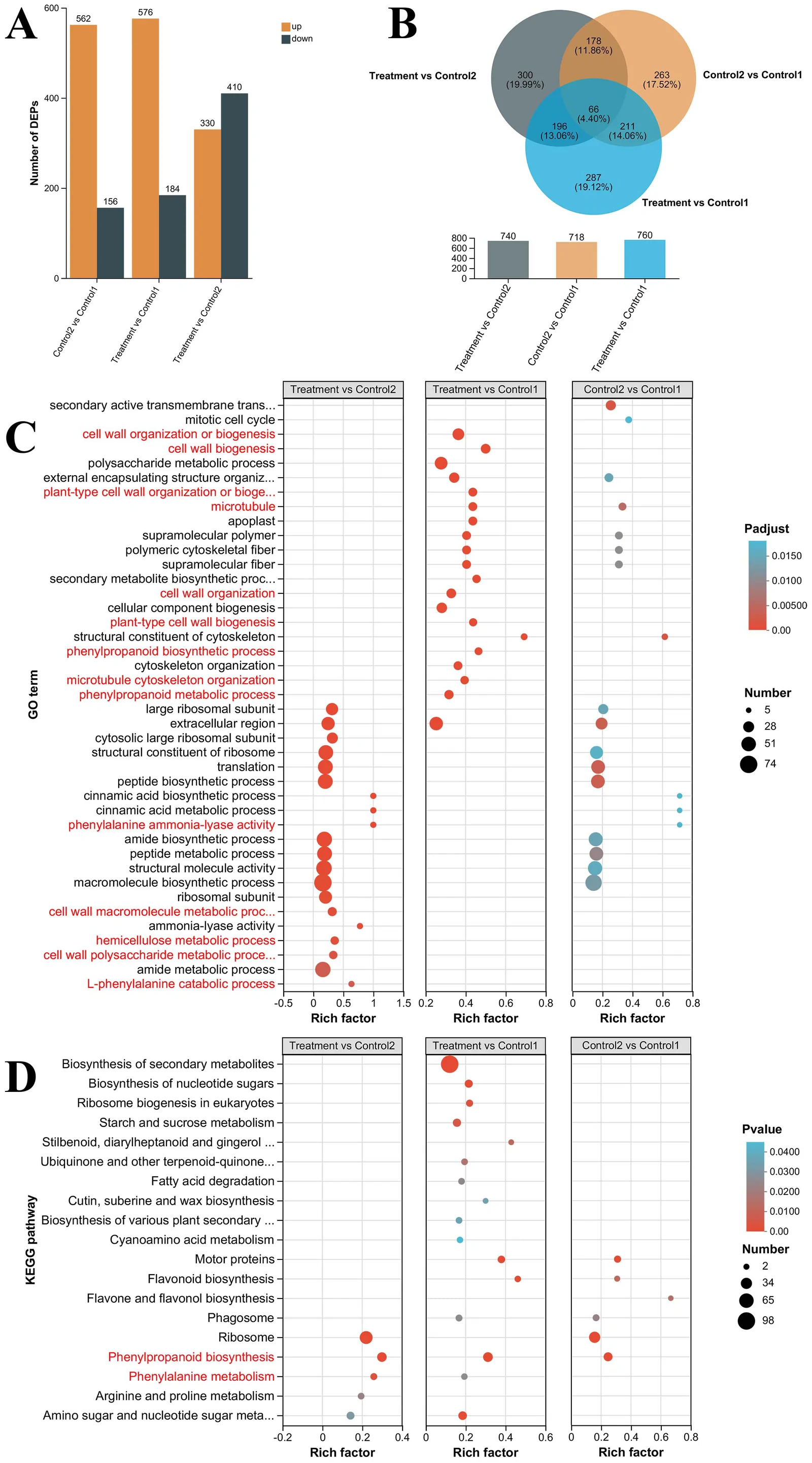
Analysis of DEPs. **(A)** Bar chart showing the number of DEPs in each comparison group, with different bar colors representing upregulated and downregulated DEPs, respectively. **(B)** Venn diagram of DEPs among comparison groups, with the total number of DEPs in each group counted. **(C, D)** GO and KEGG enrichment analyses of DEPs. In the figures, bubble color indicates the statistical significance of the enriched pathways, and bubble size represents the number of DEPs included in the corresponding pathways. n = 3 biological replicates per treatment group.

Proteomic analysis of foxtail millet samples under different fertilization treatments was performed using an Orbitrap Astral mass spectrometer, yielding a total of 90,476 peptides. Among these, 81,386 identified peptides (89.96%) consisted of 7 to 20 amino acids (**Supplementary Figure 7A**), and 8,298 proteins (89.73%) contained more than two peptides (**Supplementary Figure 7B**). Approximately 71.55% of the identified proteins (5,989) had a molecular weight ranging from 21 to 81 kDa, while 1.05% (88 proteins) exceeded 201 kDa (**Supplementary Figure 7C**). Additionally, the identified proteins exhibited excellent peptide coverage—for instance, 79.60% and 60.21% of the proteins had coverage greater than 10% and 20%, respectively (**Supplementary Figure 7D**). PCA and sample correlation heatmaps showed distinct inter-group separation and high intra-group correlation (**Supplementary Figures 8A, B**). A total of 1,501 differentially expressed proteins (DEPs) were detected across the three comparison groups, with 718, 760, and 740 DEPs identified in Control2 vs Control1, Treatment vs Control1, and Treatment vs Control2, respectively (**Figures 7A, B**).

The GO enrichment results showed that the DEPs in the Control2 vs Control1 comparison group were enriched in 30 core biological processes, including substance synthesis, metabolic processes, cellular structure construction, and cell cycle regulation. Among these, 7 terms were related to cell wall development (**Figure 7C**; **Supplementary Table 24**). In the Treatment vs Control1 group, the DEPs were enriched in 67 core biological processes, such as cellular structure construction (especially cell wall), metabolic processes (e.g., polysaccharides, secondary metabolites), and cytoskeleton organization. Notably, this group contained the most cell wall development-related terms (44 in total) among all comparison groups, covering 7 categories: cell wall organization and biogenesis, synthesis and metabolism of cell wall components (polysaccharides), synthesis and metabolism of cell wall-related substances (including precursors), key enzyme activities, structure and localization, and cytoskeleton (**Figure 7C**; **Supplementary Table 25**). For the Treatment vs Control2 group, the DEPs were enriched in 50 core biological processes, including substance synthesis, metabolic processes, and cell wall construction. A total of 24 terms were directly involved in cell wall organization and biogenesis, synthesis and metabolism of cell wall components (polysaccharides), and synthesis and metabolism of cell wall-related substances (e.g., phenols) (**Figure 7C**; **Supplementary Table 26**).

The KEGG enrichment results showed that 6, 16, and 5 pathways were significantly enriched in the Control2 vs Control1, Treatment vs Control1, and Treatment vs Control2 comparison groups, respectively. The Control2 vs Control1 group was enriched in core biological processes such as secondary metabolism, protein synthesis, cell movement, and substance transport and decomposition. The Treatment vs Control1 group was enriched in 5 pathways related to plant cell wall development, including Phenylpropanoid biosynthesis, Biosynthesis of nucleotide sugars, Amino sugar and nucleotide sugar metabolism, Phenylalanine metabolism, and Starch and sucrose metabolism. Among the 5 pathways enriched in the Treatment vs Control2 group, 3 were associated with cell wall development: Phenylpropanoid biosynthesis, Phenylalanine metabolism, and Amino sugar and nucleotide sugar metabolism (**Figure 7D**; **Supplementary Tables 27–29**).

Given that all multi-omics analyses consistently pointed to the phenylalanine pathway, we further determined the phenylalanine content in the second to fourth internodes of foxtail millet stems. The results showed that the phenylalanine content was the highest in the Control2 group, slightly lower in the Treatment group, with the two groups exhibiting increases of 24.70% and 4.85% compared to the Control1 group, respectively (**Supplementary Figure 9**). The higher phenylalanine content under compound fertilizer alone (Control2) may reflect accumulation of the substrate pool without efficient downstream flux into the phenylpropanoid pathway. Under combined fertilization (Treatment), the slightly lower phenylalanine content accompanied by upregulated *PAL*/*PTAL*/*PRDX6* and increased cell wall thickness suggests more active metabolic channeling of phenylalanine into lignin and other cell wall components. This interpretation is consistent with the multi-omics enrichment results. We constructed and analyzed a network diagram for the most significantly affected phenylalanine synthesis pathway, mapping DEGs, DAMs, and DEPs onto the network. The results showed that a total of 8 DEGs were mapped to this pathway, including 2 *PTAL* genes and 6 *PRDX6* genes. Both *PTAL* genes were upregulated in Control2 and Treatment groups, with the highest expression level in the Treatment group. Most *PRDX6* genes exhibited the same expression trend as *PTAL*, peaking in the Treatment group. Additionally, 6 metabolites showed significant differences in content, most of which were located on the G/S lignin branch. Consistent with the transcriptomic results, the content of most DAMs was the highest in the Treatment group. For DEPs, 35 were mapped to this pathway, with the main ones being PTAL, PAL, PRDX6, and their homologous proteins — echoing the transcriptomic findings. The contents of all PTAL and PAL proteins gradually increased in Control2 and Treatment groups, reaching the maximum in the Treatment group. In contrast, PRDX6 proteins displayed two expression patterns: a gradual increase or decrease sequentially across Control1, Control2, and Treatment groups.

To further integrate the multi-omics datasets, we performed pairwise Pearson correlation analysis across all 51 molecules (8 DEGs, 8 DAMs, and 35 DEPs) mapped to the phenylalanine/phenylpropanoid pathway. Each molecule was first z-score standardized (mean = 0, SD = 1) across the nine sample points (3 treatments × 3 biological replicates; for metabolomics, six technical replicates per treatment were averaged in pairs to yield three biological replicates). Among the 1,275 pairwise correlations, 449 (35.2%) were statistically significant (*p* < 0.05), and 241 (18.9%) at *p* < 0.01. Cross-omics correlations revealed considerable concordance: 41.1% (115/280) of DEG-DEP pairs, 28.2% (79/280) of DAM-DEP pairs, and 21.9% (14/64) of DEG-DAM pairs were significantly correlated (*p* < 0.05). The two *PTAL* genes (Seita.1G240200 and Seita.6G181000) showed strong positive correlations with their corresponding proteins (*r* = 0.91-0.95, *p* < 0.001), as well as with multiple downstream DEPs in the lignin biosynthesis branch. Metabolites in the G/S lignin branch (e.g., P-coumaraldehyde, coniferyl alcohol) exhibited strong correlations with peroxidase proteins, consistent with the metabolic channeling from phenylpropanoid precursors to lignin monomers. Integrative pathway enrichment scores, computed by combining KEGG enrichment *p*-values from the three omics layers using Fisher’s method, confirmed phenylpropanoid biosynthesis and phenylalanine metabolism as the top-ranked pathways associated with combined fertilization treatment (**Supplementary Figure 10**).

## 4 Discussion

**Figure 8:**
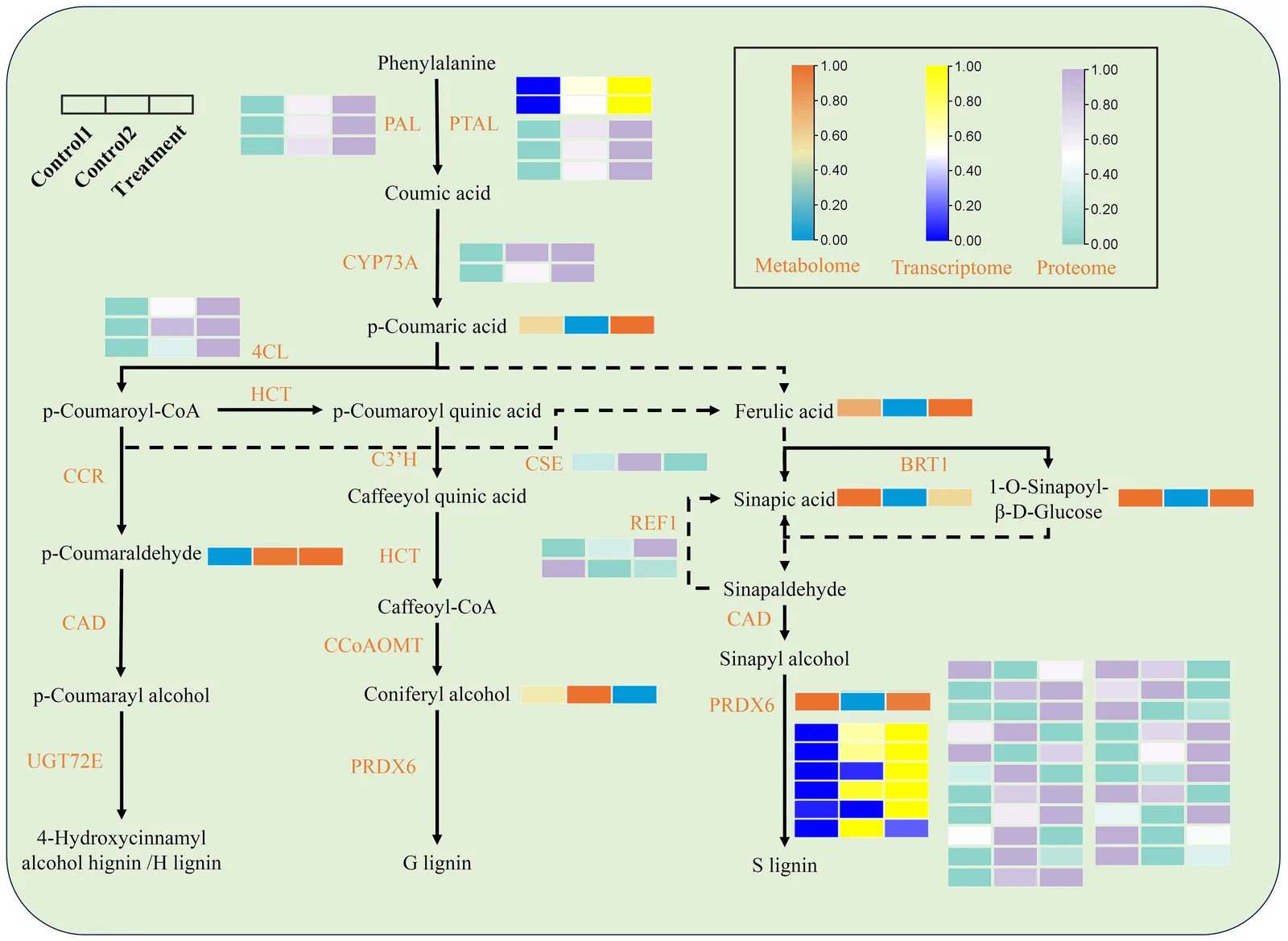
Integrative pathway map of the phenylalanine and phenylpropanoid biosynthesis pathways in foxtail millet under three fertilization treatments. DEGs (transcriptome), DAMs (metabolome), and DEPs (proteome) mapped to the pathway are shown as heatmaps alongside corresponding enzymatic steps. Transcriptome: orange (upregulated), blue (downregulated). Metabolome: yellow (upregulated), dark blue (downregulated). Proteome: lavender (upregulated), mint green (downregulated). The three columns within each heatmap correspond to Control1, Control2, and Treatment groups. n = 3 biological replicates per group per omics layer.

With its small genome and the completion of genome mapping, foxtail millet has increasingly become a model plant for studying the genetic mechanisms of gramineous plants (Bennetzen et al., 2012; Zhang et al., 2012; He et al., 2024). Meanwhile, the development of high-throughput sequencing technology has provided a convenient technical means for analyzing various environmental response patterns of foxtail millet through integrated multi-omics analysis, among which the exploration of nutrient response patterns of foxtail millet is a key research direction (Meng et al., 2024; Yao et al., 2024). Different from other studies focusing on the yield of foxtail millet and the quality of millet, this study concentrated on the effects of fertilizers on plant stems, especially the development of cell walls, and specifically analyzed the potential relationship between fertilizers and stem structural properties (vascular area, cell wall thickness, and puncture strength) that are relevant to but not equivalent to lodging resistance. Dedicated field lodging assays would be required to establish a direct link to lodging resistance. At present, the research on lodging resistance of foxtail millet mainly calculates the lodging index through traditional field experiments, lacking studies on cell wall development which is directly related to lodging resistance (Tian et al., 2017; Zhong et al., 2024). However, the development degree of cell wall is one of the direct factors affecting the lodging resistance of foxtail millet. The phenylalanine pathway is an important pathway affecting plant cell wall development, which is associated with cell wall development by influencing lignin, one of the main components of cell wall (Fan et al., 2018; Geng et al., 2020; Guo et al., 2024). Therefore, this pathway plays an irreplaceable role in the research on secondary cell wall development of woody plants (Shang et al., 2022; Du et al., 2024). Since wood is not the main harvest target of gramineous plants, the research on cell wall of gramineous plants is relatively limited. In the research of foxtail millet, it has been found that the changes in the expression levels of genes in pathways such as phenylalanine can cause changes in the plant height of foxtail millet and even affect its fertility (Gao et al., 2022; Li et al., 2025). For example, the mutation of the boron transporter *BOR1* gene in foxtail millet upregulates the expression of genes such as *PAL* in the phenylalanine pathway, and the cell wall thickness of the mutant is greater (Wang et al., 2022). The role of *PAL* has been implicated in cell wall deposition in other plant systems (Cass et al., 2015; Muthamilarasan et al., 2015). However, in the present study, the observed correlations between *PAL*/*PTAL*/*PRDX6* expression and cell wall phenotypes are correlative. Functional validation through genetic approaches (e.g., CRISPR/Cas9 mutagenesis or overexpression) or enzyme activity assays would be needed to confirm their direct involvement in stem strengthening in foxtail millet. In our transcriptomic results, only the expression levels of *PAL* homologous genes *PTAL* and peroxidase changed, almost all showing an upward trend along Control1-Control2-Treatment, and this trend was maintained in their translated proteins (**Figure 8**). Coordinately, the application of compound fertilizer increased the content of endogenous phenylalanine and the thickness of cell wall (**Supplementary Figure 7A**). Here, there is a question worthy of discussion, that is, the exploration of the reasons for the increase of phenylalanine content, which may involve the effect of compound fertilizer (or the combined application of cattle manure-based organic fertilizer and compound fertilizer) on phenylalanine synthesis. Existing literatures have provided interesting perspectives: studies have found that cow manure is rich in cellulose, and Bacillus involved in cellulose decomposition can be isolated from various cow manures (Yang et al., 2023; Cao et al., 2024; Demissie et al., 2024). Based on this, we conducted an integrated multi-omics analysis (transcriptomics, metabolomics, and proteomics) on foxtail millet plants treated with cattle manure-based organic fertilizer and compound fertilizer, aiming to elucidate the effects of different fertilization treatments on foxtail millet stem growth and development. Combined phenotypic measurements and multi-omics analyses showed that, compared with the sole application of organic manure, compound fertilizer application promoted the expression or content changes of genes, metabolites, and proteins involved in cell wall development-related enrichment terms. At the metabolic pathway level, these changes were ultimately concentrated on the phenylalanine pathway, manifesting as variations in the synthesis and metabolism of related genes, metabolites, and proteins in this pathway. Moreover, such changes were more significant when cattle manure-based organic fertilizer and compound fertilizer were applied in combination. The multi-omics results from the combined fertilization treatment preliminarily revealed that the co-application of cattle manure-based organic fertilizer and compound fertilizer affects the biosynthesis of the phenylalanine synthesis pathway, thereby ultimately regulating the stem cell wall development of foxtail millet.

As an easily accessible traditional farmyard manure, cow manure holds significant research value in improving soil organic matter content, enhancing plant tolerance to abiotic stresses, and realizing the organic substitution of chemical fertilizers (Pan and Huang, 2024; Zhou et al., 2025). However, current studies on the effects of cow manure on plant endogenous or exogenous hormones remain relatively insufficient. In this study, we found that compared with the compound fertilizer treatment, the upregulated differentially expressed genes (DEGs) in foxtail millet after cattle manure-based organic fertilizer treatment were enriched in indole-3-acetic acid (IAA)-related terms, including the metabolism and processes of tryptophan and indole-containing compounds. Meanwhile, the KEGG enrichment results were also enriched in pathways related to IAA synthesis, namely Indole alkaloid biosynthesis and Tryptophan metabolism (Rosquete et al., 2012). Therefore, we speculate that the application of cattle manure-based organic fertilizer may affect the synthesis of endogenous auxin, and existing studies have supported this speculation. The NEAU-LLBT strain (belonging to Microbacterium stercoris sp. nov) isolated from cow manure in Shangzhi City, Heilongjiang Province, is considered to be potentially involved in the synthesis of plant endogenous auxin (Zhang et al., 2021). We also determined the endogenous auxin content in foxtail millet stem segments. As expected, the IAA content in the stem segments of foxtail millet in the cattle manure-based organic fertilizer treatment group (Control1) was significantly higher than that in the other two treatment groups, with the average values being 13.15% and 14.99% higher, respectively (**Supplementary Figure 7B**). On the one hand, this confirms the reliability of the transcriptomic results; on the other hand, the results provide us with an insight that the application of cattle manure-based organic fertilizer may also affect the level of plant endogenous hormones. This also gives us a positive prospect for the application of cattle manure-based organic fertilizer: elucidating the effect of cattle manure-based organic fertilizer application on the endogenous hormones of foxtail millet may improve the field utilization rate of cattle manure-based organic fertilizer and reduce the use of growth regulators. In the future, we can also take this as an entry point to conduct in-depth research, explore the specific mechanisms by which different fertilization modes affect hormone contents, and analyze the effects of soil microorganisms on hormones and growth of *Setaria italica*.

Future research directions: Several hypotheses emerging from this study warrant further investigation. First, cattle manure application is known to alter soil microbial community composition and diversity (Fan et al., 2023; Sagar et al., 2023); we speculate that microbially derived signals or metabolites could potentially influence phenylalanine metabolism in foxtail millet, though no soil microbiome or rhizosphere analyses were performed in this study. Second, the potential involvement of IAA-related signaling in mediating fertilizer effects on stem growth remains an intriguing but untested hypothesis. Dedicated experiments incorporating soil metagenomics, rhizosphere metabolomics, and hormone profiling are needed to evaluate these possibilities.

Several limitations of this study should be acknowledged. First, the experimental design included three fertilization strategies (organic, inorganic, and combined) but did not include an unfertilized (zero-fertilizer) control. Therefore, this study compares the relative effects of different fertilization strategies rather than the absolute effects of fertilization per se. Second, the anatomical and mechanical measurements (vascular area, cell wall thickness, stem puncture strength) are indirect indicators of lodging-related traits; dedicated field lodging assays under natural or artificially induced lodging conditions are needed to directly assess lodging resistance. Third, the conclusions regarding the phenylalanine pathway are based on correlative multi-omics evidence; functional validation through genetic or biochemical approaches is required to establish causal relationships.

## 5 Conclusion

This study employed an integrated multi-omics approach (transcriptomics, metabolomics, and proteomics) to investigate the effects of different fertilization strategies on stem development in foxtail millet. Combined phenotypic measurements and multi-omics analyses showed that, compared with sole application of organic manure, combined application of cattle manure-based organic fertilizer and compound fertilizer was associated with enhanced stem structural properties, including increased vascular area, cell wall thickness, and stem puncture strength. Multi-omics analyses consistently identified the phenylalanine metabolism and phenylpropanoid biosynthesis pathways as being enriched across treatments, with the strongest enrichment observed under combined fertilization. Pairwise Pearson correlation analysis across all 51 pathway-mapped molecules revealed substantial cross-omics concordance, with PTAL genes and their corresponding proteins showing particularly strong co-variation. These findings suggest that the phenylalanine pathway is a candidate pathway associated with stem cell wall development under combined fertilization, though functional validation is needed to establish causal relationships. This study provides a theoretical basis and multi-omics resource for optimizing fertilization strategies in foxtail millet cultivation.

## Data Availability

The datasets presented in this study can be found in online repositories. The names of the repository/repositories and accession number(s) can be found in the article/**Supplementary Material**. Transcriptomic data have been deposited in the Genome Sequence Archive (GSA) under accession CRA030049; metabolomic data have been deposited in OMIX under accession OMIX011888; proteomic data have been deposited in OMIX under accession OMIX012862.

## Supplementary material

Supplementary Figure 1 Primer information of each gene in qPCR.

Supplementary Figure 2 PCA and sample correlation analysis of transcriptomic sequencing data. **(A)** PCA analysis. **(B)** Sample correlation analysis, where the correlation between samples is represented by the color and area of pie charts.

Supplementary Figure 3 qPCR validation of transcriptomic results. Expression profiles of ten selected genes across three treatment groups (Control1, Control2, Treatment) measured by RNA-seq (black lines, left Y-axis, TPM) and qPCR (red lines, right Y-axis, relative expression level, REL). Data are presented as means ± SE (n = 3 biological replicates per treatment group).

Supplementary Figure 4 Validation of RNA-seq expression by qPCR. **(A)** Scatter plot of pooled Z-score-normalized RNA-seq and qPCR expression values across 10 genes from three treatment groups, with three biological replicates per group (n = 90 data points). The dashed line represents the linear regression, and the pooled Pearson correlation coefficient (*r*) and two-tailed *p*-value are indicated. **(B)** Per-gene Pearson correlation coefficients between RNA-seq and qPCR expression values (n = 9 per gene).

Supplementary Figure 5 Information of metabolic samples and DAMs. **(A)** PCA analysis. **(B)** Sample correlation analysis. **(C)** PLS-DA permutation test. **(D)** Classification information of DAMs. **(E–G)** Classification information of DAMs in each comparison group. n = 6 technical replicates per treatment group.

Supplementary Figure 6 Human Metabolome Database (HMDB) classification of the top 60 DAMs from VIP analysis. **(A–C)** Represent the comparison groups of Control2 vs Control1, Treatment vs Control1, and Treatment vs Control2, respectively.

Supplementary Figure 7 Distribution of peptides and proteins. **(A)** Statistics of peptide length. **(B)** Statistics of peptide count. **(C)** Molecular weight distribution of proteins. **(D)** Coverage distribution of proteins. n = 3 biological replicates per treatment group.

Supplementary Figure 8 PCA and sample correlation analysis of proteomic data. **(A)** Principal component analysis. **(B)** Sample correlation heatmap. n = 3 biological replicates per treatment group.

Supplementary Figure 9 Determination of endogenous phenylalanine and indole-3-acetic acid (IAA) contents in foxtail millet stems. **(A)** Phenylalanine content. **(B)** IAA content. Data are presented as means ± SE (n = 3 biological replicates). Different letters above bars indicate significant differences among treatments (one-way ANOVA with Dunnett’s test, *p* < 0.05).

Supplementary Figure 10 Multi-omics correlation network of molecules mapped to the phenylalanine/phenylpropanoid pathway. Pairwise Pearson correlation coefficients were calculated across all 51 molecules (8 DEGs, red; 8 DAMs, green; 35 DEPs, blue) after z-score standardization (mean = 0, SD = 1) across nine sample points (3 treatments × 3 biological replicates). Open circles denote significant correlations (*p* < 0.05, two-tailed). PTAL genes and their corresponding proteins (r = 0.91–0.95) and G/S lignin-branch metabolites with peroxidase proteins (|*r*| > 0.9) represent the strongest co-variation patterns, consistent with coordinated regulation across transcript, metabolite, and protein levels.

## References

[B1] Adesemoye A. O. Torbert H. A. Kloepper J. W. (2009). Plant growth-promoting rhizobacteria allow reduced application rates of chemical fertilizers. Microb. Ecol. 58, 921–929. doi: 10.1007/s00248-009-9531-y 19466478

[B2] Bennetzen J. L. Schmutz J. Wang H. Percifield R. Hawkins J. Pontaroli A. C. . (2012). Reference genome sequence of the model plant Setaria. Nat. Biotechnol. 30, 555–561. doi: 10.1038/nbt.2196 22580951

[B3] Cao X. Li L. Zhang F. Zhang F. Song X. Zhao W. . (2024). Green development of natural fibre-based paper mulch from recyclable cow dung and flax straw waste. Agronomy-Basel 14, 290. doi: 10.3390/agronomy14020290 30654563

[B4] Cass C. L. Peraldi A. Dowd P. F. Mottiar Y. Santoro N. Karlen S. D. . (2015). Effects of PHENYLALANINE AMMONIA LYASE (PAL) knockdown on cell wall composition, biomass digestibility, and biotic and abiotic stress responses in Brachypodium. J. Exp. Bot. 66, 4317–4335. doi: 10.1093/jxb/erv269 26093023 PMC4493789

[B5] Chen R. Chang H. Wang Z. Lin H. (2023). Determining organic-inorganic fertilizer application threshold to maximize the yield and quality of drip-irrigated grapes in an extremely arid area of Xinjiang, China. Agric. Water Manage. 276, 108070. doi: 10.1016/j.agwat.2022.108070 38826717

[B6] Dai K. Wang X. Liu H. Qiao P. Wang J. Shi W. . (2024). Efficient identification of QTL for agronomic traits in foxtail millet (Setaria italica) using RTM- and MLM-GWAS. Theor. Appl. Genet. 137, 18. doi: 10.1007/s00122-023-04522-8 38206376

[B7] Demissie M. S. Legesse N. H. Tesema A. A. (2024). Isolation and characterization of cellulase producing bacteria from forest, cow dung, Dashen brewery and agro-industrial waste. PloS One 19, e0301607. doi: 10.1371/journal.pone.0301607 38598514 PMC11006139

[B8] Department of Rural Surveys, N.S.O.C (2023). China Yearbook of Agricultural Product Price Survey - 2023 (Beijing: China Statistics Press).

[B9] Du J. Wei H. Song X. Zhang L. Hu J. (2024). PdRabG3f interfered with gibberellin-mediated internode elongation and xylem developing in poplar. Plant Sci. 343, 112074. doi: 10.1016/j.plantsci.2024.112074 38548138

[B10] Fan C. Li Y. Hu Z. Hu H. Wang G. Li A. . (2018). Ectopic expression of a novel OsExtensin‐like gene consistently enhances plant lodging resistance by regulating cell elongation and cell wall thickening in rice. Plant Biotechnol. J. 16, 254–263. doi: 10.1111/pbi.12766 28574641 PMC5785348

[B11] Fan Y. Lv G. Chen Y. Chang Y. Li Z. (2023). Differential effects of cow dung and its biochar on Populus euphratica soil phosphorus effectiveness, bacterial community diversity and functional genes for phosphorus conversion. Front. Plant Sci. 14. doi: 10.3389/fpls.2023.1242469 37780507 PMC10538999

[B12] Gao S. Chen J. Xu L. (2020). Study on drought zone division in Huang-Huai-Hai area. Water Saving Irrigation, 101–106. doi: 10.3969/j.issn.1007-4929.2020.10.021

[B13] Gao Y. Yuan Y. Zhang X. Song H. Yang Q. Yang P. . (2022). Conuping BSA-Seq and RNA-Seq reveal the molecular pathway and genes associated with the plant height of foxtail millet (Setaria italica). Int. J. Mol. Sci. 23, 11824. doi: 10.3390/ijms231911824 36233125 PMC9569614

[B14] Geng P. Zhang S. Liu J. Zhao C. Wu J. Cao Y. . (2020). MYB20, MYB42, MYB43, and MYB85 regulate phenylalanine and lignin biosynthesis during secondary cell wall formation. Plant Physiol. 182, 1272–1283. doi: 10.1104/pp.19.01070 31871072 PMC7054866

[B15] Guo F. Yu W. Fu F. Hou H. Zhang J. Guo J. . (2024). Ginkgo biloba wood transcriptome reveals critical genes for secondary cell wall formation and transcription factors involved in lignin biosynthesis. Ind. Crop Prod. 216, 118736. doi: 10.1016/j.indcrop.2024.118736 38826717

[B16] He Q. Wang C. He Q. Zhang J. Liang H. Lu Z. . (2024). A complete reference genome assembly for foxtail millet and Setaria-db, a comprehensive database for Setaria. Mol. Plant 17, 219–222. doi: 10.1016/j.molp.2023.12.017 38155573

[B17] Hongo S. Sato K. Yokoyama R. Nishitani K. (2012). Demethylesterification of the primary wall by PECTIN METHYLESTERASE35 provides mechanical support to the Arabidopsis stem. Plant Cell 24, 2624–2634. doi: 10.1105/tpc.112.099325 22693281 PMC3406921

[B18] Klinger K. M. Liebner F. Fritz I. Potthast A. Rosenau T. (2013). Formation and ecotoxicity of N-heterocyclic compounds on ammoxidation of mono- and polysaccharides. J. Agric. Food. Chem. 61, 9004–9014. doi: 10.1021/jf4019596 23967874 PMC3788623

[B19] Li P. Brutnell T. P. (2011). Setaria viridis and Setaria italica, model genetic systems for the Panicoid grasses. J. Exp. Bot. 62, 3031–3037. doi: 10.1093/jxb/err096 21459768

[B20] Li Z. D. Yao X. L. Zhang L. Zhang J. Hao J. H. Wu H. . (2025). Transcriptome profiling reveals abnormal cell wall components in the cleistogamy mutant 1 (clm1) lodicule of foxtail millet. Planta 262. doi: 10.1007/s00425-025-04722-0 40394337

[B21] Ma Y. Liu W. Qiao Y. Qiao W. Yang H. Zhong Y. . (2023). Effects of soil salinity on foxtail millet osmoregulation, grain yield, and soil water utilization under varying water conditions. Agric. Water Manage. 284, 108354. doi: 10.1016/j.agwat.2023.108354 38826717

[B22] Meng H. X. Wang Y. Z. Yao X. L. Xie X. R. Dong S. Yuan X. Y. . (2024). Reactive oxygen species (ROS) modulate nitrogen signaling using temporal transcriptome analysis in foxtail millet. Plant Mol. Biol. 114. doi: 10.1007/s11103-024-01435-y 38602592

[B23] Muthamilarasan M. Khan Y. Jaishankar J. Shweta S. Lata C. Prasad M. (2015). Integrative analysis and expression profiling of secondary cell wall genes in C_4_ biofuel model Setaria italica reveals targets for lignocellulose bioengineering. Front. Plant Sci. 6. doi: 10.3389/fpls.2015.00965 26583030 PMC4631826

[B24] Ni X. Xia Q. Zhang H. Cheng S. Li H. Fan G. . (2017). Updated foxtail millet genome assembly and gene mapping of nine key agronomic traits by resequencing a RIL population. GigaScience 6, 1–8. doi: 10.1093/gigascience/giw005 28369461 PMC5466707

[B25] Pan C. C. Huang C. H. (2024). Cow dung compost and vermicompost amendments promote soil carbon stock by enhancing labile organic carbon and residual oxidizable carbon fractions in maize field soil. Soil Use Manage. 40. doi: 10.1111/sum.13122 40046247

[B26] Pan J. Li Z. Wang Q. Garrell A. K. Liu M. Guan Y. . (2018). Comparative proteomic investigation of drought responses in foxtail millet. BMC Plant Biol. 18, 315. doi: 10.1186/s12870-018-1533-9 30497407 PMC6267058

[B27] Qu Y. Chen X. Gao H. Lyu J. Su Z. (2023). An analysis of distribution characteristics of agricultural drought disaster risks in China. China Water Resour., 23–27. doi: 10.3969/j.issn.1000-1123.2023.08.007

[B28] Rajyaguru R. H. Maheshala N. Padiya P. S. Bhalani H. Tomar R. S. (2024). Genetic Improvement of Foxtail Millet Through Advanced Biotechnological Methods (Singapore: Springer), 365–382.

[B29] Ren Y. Yu G. Shi C. Liu L. Guo Q. Han C. . (2022). Majorbio Cloud: A one‐stop, comprehensive bioinformatic platform for multiomics analyses. Imeta 1, e12. doi: 10.1002/imt2.12 38868573 PMC10989754

[B30] Rengasamy P. (2006). World salinization with emphasis on Australia. J. Exp. Bot. 57, 1017–1023. doi: 10.1093/jxb/erj108 16510516

[B31] Rosquete M. R. Barbez E. Kleine-Vehn J. (2012). Cellular auxin homeostasis: gatekeeping is housekeeping. Mol. Plant 5, 772–786. doi: 10.1093/mp/ssr109 22199236

[B32] Sagar S. Singh A. Bala J. Chauhan R. Kumar R. Bhatia R. K. . (2023). Insights into cow dung-based bioformulations for sustainable plant health and disease management in organic and natural farming system: a review. J. Soil Sci. Plant Nutr. 24, 30–53. doi: 10.1007/s42729-023-01558-z 30311153

[B33] Shang B. Tian T. Mo Y. Zhang H. Zhang K. Agathokleous E. . (2025). Combined application of organic and inorganic fertilizers sustained rice yields and N accumulation and decreased soil-canopy system NH_3_ emission. Agric. Ecosyst. Environ. 377, 109260. doi: 10.1016/j.agee.2024.109260 38826717

[B34] Shang X. Zhang P. Liu G. Zhan N. Wu Z. (2022). Comparative transcriptomics analysis of contrasting varieties of Eucalyptus camaldulensis reveals wind resistance genes. PeerJ 10, e12954. doi: 10.7717/peerj.12954 35233295 PMC8882336

[B35] Tian B. Liu Y. Zhang L. Li H. (2017). Stem lodging parameters of the basal three internodes associated with plant population densities and developmental stages in foxtail millet (Setaria italica) cultivars differing in resistance to lodging. Crop Pasture Sci. 68, 349. doi: 10.1071/cp16453 38477348

[B36] Wan X. F. Wang S. Zhang Y. Wang T. L. Ge Y. Gao S. X. . (2021). Effect of combined application of inorganic and organic fertilizers on growth and quality of Salvia miltiorrhiza. China J. Chin. Materia Med. 46, 1927–1934. doi: 10.19540/j.cnki.cjcmm.20210205.101 33982501

[B37] Wang H. Tang S. Zhi H. Xing L. Zhang H. Tang C. . (2022). The boron transporter SiBOR1 functions in cell wall integrity, cellular homeostasis, and panicle development in foxtail millet. Crop J. 10, 342–353. doi: 10.1016/j.cj.2021.05.002 38826717

[B38] Yang C. Yang L. Ouyang Z. (2005). Organic carbon and its fractions in paddy soil as affected by different nutrient and water regimes. Geoderma 124, 133–142. doi: 10.1016/j.geoderma.2004.04.008 38826717

[B39] Yang J. Ren Y. Jia M. Huang S. Guo T. Liu B. . (2025). Improving soil quality and crop yield of fluvo-aquic soils through long-term organic-inorganic fertilizer combination: Promoting microbial community optimization and nutrient utilization. Environ. Technol. Innov. 37, 104050. doi: 10.1016/j.eti.2025.104050 38826717

[B40] Yang X. Li L. Zhao W. Wang M. Yang W. Tian Y. . (2023). Characteristics and functional application of cellulose fibers extracted from cow dung wastes. Materials 16, 648. doi: 10.3390/ma16020648 36676384 PMC9866732

[B41] Yao X. L. Wang Y. Z. Meng H. X. Zhang M. H. Zhou X. Kang X. T. . (2024). Identification of systemic nitrogen signaling in foxtail millet (Setaria italica) roots based on split-root system and transcriptome analysis. Plant Cell Rep. 43, 243. doi: 10.1007/s00299-024-03338-0 39340664

[B42] Zavattaro L. Assandri D. Grignani C. (2016). Achieving legislation requirements with different nitrogen fertilization strategies: Results from a long term experiment. Eur. J. Agron. 77, 199–208. doi: 10.1016/j.eja.2016.02.004 38826717

[B43] Zhang L. Jiao Y. Ling L. Wang H. Song W. Zhao T. . (2021). Microbacterium stercoris sp. nov. an indole acetic acid-producing actinobacterium isolated from cow dung. Int. J. Syst. Evol. Microbiol. 71. doi: 10.1099/ijsem.0.005099 34762581

[B44] Zhang G. Liu X. Quan Z. Cheng S. Xu X. Pan S. . (2012). Genome sequence of foxtail millet (Setaria italica) provides insights into grass evolution and biofuel potential. Nat. Biotechnol. 30, 549–554. doi: 10.1038/nbt.2195 22580950

[B45] Zhong Y. Zhang T. Qiao W. Liu W. Qiao Y. Li Y. . (2024). Optimizing canopy spacing configuration enhances foxtail millet grain yield and water productivity by improving stalk lodging resistance in the North China Plain. Eur. J. Agron. 158, 127230. doi: 10.1016/j.eja.2024.127230 38826717

[B46] Zhou H. Yu Z. Zhang S. Zong Q. Zhang Y. Pang Y. . (2025). Mitigating secondary salinization in grapes: long-term benefits of biochar and cow dung. Front. Plant Sci. 16. doi: 10.3389/fpls.2025.1528354 40093615 PMC11906475

